# Lanatoside C Promotes Foam Cell Formation and Atherosclerosis

**DOI:** 10.1038/srep20154

**Published:** 2016-01-29

**Authors:** Huairui Shi, Xiaobo Mao, Yucheng Zhong, Yuzhou Liu, Xiaoqi Zhao, Kunwu Yu, Ruirui Zhu, Yuzhen Wei, Jianghao Zhu, Haitao Sun, Yi Mao, Qiutang Zeng

**Affiliations:** 1 The Laboratory of Cardiovascular Immunology, Institute of Cardiology, Union Hospital, Tongji Medical College, Huazhong University of Science and Technology, Wuhan, China

## Abstract

Lanatoside C’s impact on atherosclerosis is poorly understood. The present study was conducted to determine whether lanatoside C affects the development of atherosclerosis in apolipoprotein E-deficient (ApoE^–/–^) mice. ApoE^–/–^ mice were administered either phosphate-buffered saline (PBS) containing 0.1% DMSO (the vehicle control group) or lanatoside C at low (1 mg/kg per day) or high (2 mg/kg per day) doses, and fed a Western diet for 12 weeks. Lanatoside C dose-dependently aggravated the development of atherosclerosis in the ApoE^–/–^ mice compared with the vehicle control group. In an effort to determine the mechanism by which lanatoside C increased atherosclerosis, we found that lanatoside C significantly promoted the uptake of oxidised low-density lipoprotein (oxLDL) and increased foam-cell formation by upregulation of scavenger receptor class A (SR-A) and the class B scavenger receptor (CD36) in macrophages. Meanwhile, the effects of lanatoside C were abolished using small interfering RNA (siRNA) inhibition of peroxisome proliferator-activated receptors β/δ (PPARβ/δ). Overall, our data demonstrate that lanatoside C aggravates the development of atherosclerosis by inducing PPARβ/δ expression, which mediates upregulation of SR-A and CD36, and promotes oxLDL uptake and foam-cell formation.

Atherosclerosis is a chronic disease of the large arteries that is an important cause of morbidity and mortality in industrialised nations^1^,^2^. Lipoprotein uptake by monocyte-derived macrophages is thought to be one of the earliest pathogenic events in the nascent plaque, and results in the development of early foam-cell formation^3^,^4^. The role of foam cells as the major culprit in atherosclerosis has been further demonstrated by the resistance to atherosclerosis in ApoE^–/–^ mice^5^,^6^. This unrestricted uptake through scavenger receptor pathways, which is not limited by intracellular cholesterol levels, eventually leads to the formation of foam cells, the initial step in atherosclerosis^7^,^8^. Foam-cell formation is increased by several extracellular factors, especially uncontrolled uptake of oxidised low-density lipoprotein (oxLDL) that exceeds cholesterol influx, subsequently triggering the formation of foam cells^9^.

The intracellular lipid homeostasis of macrophages is dynamically regulated by oxLDL uptake and cholesterol efflux. Macrophage scavenger receptor class A (SR-A) and CD36, a member of the type B family, are thought to play significant roles in foam-cell formation because of their ability to promote uptake-modified lipids, such as oxLDL^7^,^10^. The absence of CD36 and SR-A reduced 75%–90% of oxLDL uptake internalization by macrophages in some studies^11^,^12^,^13^. The removal of cellular cholesterol is critical for preventing foam-cell formation and the development of atherosclerotic lesions^14^,^15^,^16^. ATP-binding cassette (ABC) transporters (ABCA1 and ABCG1) and SR-BI, another type B scavenger receptor, protect against foam-cell formation when expressed in macrophages, by stimulating cholesterol efflux^17^,^18^,^19^.

The nuclear receptor subfamily of the peroxisome proliferator-activated receptors (PPARs) family consists of α (NR1C1), β/δ (NR1C3) and γ (NR1C2) isoforms^20^,^21^,^22^,^23^. PPARs exert profound effects on the metabolism of lipoproteins, fatty acids and inflammatory responses^24^,^25^,^26^,^27^,^28^. PPARβ/δ is expressed in many tissues, particularly the gut, kidneys and heart. PPARβ/δ increases lipid accumulation by increasing expression of SR-A and CD36^29^,^30^. Cheng published a paper showing that cardiac glycoside digoxin increases PPARβ/δ expression in H9c2 cells^31^. Lanatoside C, as a US Food and Drug Administration (FDA)-approved cardiac glycoside, is used in the treatment of congestive heart failure and cardiac arrhythmia, and recent studies have found that lanatoside C also inhibits several negative-strand RNA viruses^32^,^33^. However, its impact on atherosclerosis is poorly understood. The present study was conducted to determine whether the cardiac glycoside lanatoside C affects the development of atherosclerosis in ApoE^–/–^ mice.

## Results

### Lanatoside C aggravates atherosclerosis development in ApoE^–/–^ mice

**Figure 1:**
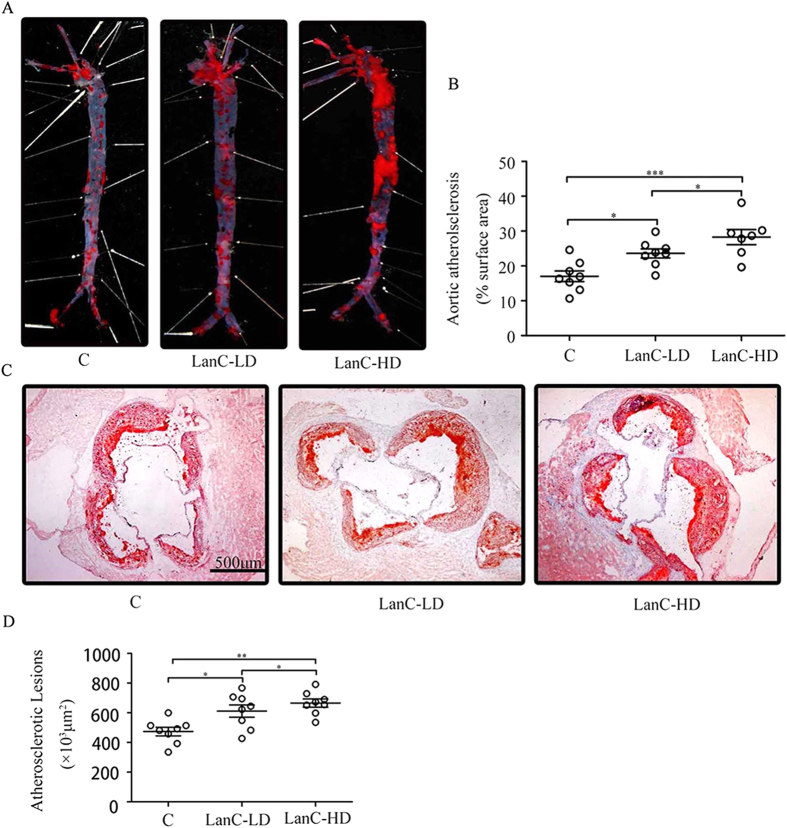
Lanatoside C promotes the development of atherosclerosis in ApoE^–/–^ mice. (**A**) Representative images of Oil Red O staining of *en face* preparations of the aortas in the different treatment groups (low-dose lanatoside C, 1 mg/kg per day; high-dose lanatoside C, 2 mg/kg per day) or vehicle control (PBS containing 0.1% DMSO). (**B**) Quantitative analysis of the atherosclerotic surface area of the entire aorta. (**C**) Cryosections of the aortic sinus stained with Oil Red O, with hematoxylin used as a counterstain. (**D**) The lesion sizes in the aortic roots were averaged to determine the lesion size of ten sections of the aortic sinus. The data are expressed as mean ± SEM. One-way ANOVA was followed by the Holm-Sidak test. *P ≤ 0.05; **P ≤ 0.01; ***P ≤ 0.001; C, n = 8; LanC-LD, n = 8; LanC-HD, n = 8; C (vehicle control); LanC-LD (low-dose lanatoside C); LanC-HD (high-dose lanatoside C).

The ApoE^−/−^ mouse is a well-established animal model for studying atherosclerosis^34^,^35^,^36^. Our preliminary experiment showed that the different doses of lanatoside C caused various toxic reactions and death. ApoE^–/–^ mice were given different doses of lanatoside C and observed for 48 h. A higher dose (3 mg/kg per day and 4 mg/kg per day) could increase the risk of toxic reaction and death (Table S1), but not 2mg/kg per day. To investigate the potential effects of lanatoside C on atherosclerosis, we evaluated the severity of atherosclerosis based on the morphological and histological changes that occurred in mice treated with approximately 20 μg of lanatoside C (low-dose, 1 mg/kg per day) or 40 μg of lanatoside C (high-dose, 2 mg/kg per day), compared to the vehicle control (PBS containing 0.1% DMSO). After the 12-week lanatoside C treatment, lanatoside C levels were measured 24 h following the final injection (low-dose lanatoside C, 0.9 ± 0.10 ng/ml; high-dose lanatoside C, 2.2 ± 0.15 ng/ml) (Fig. S1). Lanatoside C aggravated the development of atherosclerosis (Fig. 1A), and the extent of atherosclerosis in the mice receiving 2 mg/kg of lanatoside C was approximately 60% greater than in the vehicle control mice (Fig. 1A,C). Quantification of the Oil Red O staining of the entire aorta revealed accelerated atherosclerotic plaque formation in mice treated with different doses of lanatoside C when compared with the vehicle control-treated group (low-dose lanatoside C, 23.52 ± 1.33%; high-dose lanatoside C, 29.72 ± 1.46%; vehicle control, 16.93 ± 1.73%) (Fig. 1B). These findings correlate with the amounts of atherosclerotic lesions noted in the aortic sinuses of mice treated with low and high doses of lanatoside C or the vehicle control (611.48 ± 53.43 and 672.43 ± 78.72 × 10^3^ μm^2^ versus 497.29 ± 106.58 × 10^3^ μm^2^, respectively) (Fig. 1D).

### Metabolic effects of lanatoside C

To assess the potentially atherosclerosis-promoting effects of lanatoside C on lipid metabolism, we monitored plasma lipids in ApoE^–/–^ mice during the treatment period. Blood samples were collected retro-orbitally on days 0, 30, 60 and 90 of the study. Total cholesterol, LDL cholesterol, HDL cholesterol and triglyceride levels were measured, and as shown in Table 1, these concentrations in the ApoE^–/–^ mice on the chow diet were approximately 310 mg/dL, 165 mg/dL, 201 mg/dL and 125 mg/dL, respectively. After being fed an atherogenic diet for 12 weeks, these levels increased to approximately 1120 mg/dL, 400 mg/dL, 865 mg/dL and 220 mg/dL, respectively. On repeated-measures ANOVA, no significant changes were observed in the plasma lipids between the treatment groups.

### Lanatoside C induces SR-A and CD36 expression in ApoE^–/–^ mice

**Figure 2:**
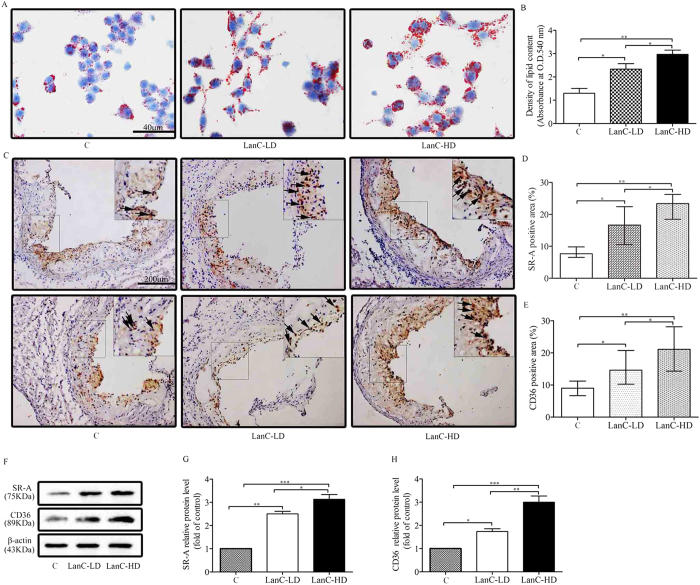
Effect of lanatoside C on foam-cell formation and scavenger receptors in ApoE^–/–^ mice. (**A**) *In vivo* foam-cell formation in the lanatoside C-treated mice (n = 5 per group). Oil Red O staining of peritoneal macrophages isolated from mice treated with lanatoside C for 12 weeks (low-dose lanatoside C, 1 mg/kg per day; high-dose lanatoside C, 2 mg/kg per day) or vehicle control (PBS containing 0.1% DMSO). **(B**) The stained cells were eluted with isopropanol, the supernatant was collected, and the optical density of the extracts was measured at 540 nm. (**C**) Representative photomicrographs of aortic root sections stained with SR-A and CD36 (n = 8 per group). (**D**,**E**) Quantitative analysis of data. (**F**–**H**) Western blot for SR-A and CD36 in aorta samples. ApoE^–/–^ mice treated with lanatoside C for 12 weeks (n = 5 per group). The fold-change in protein expression in the treated cells relative to the vehicle control-treated cells was determined (after normalization to β-actin) based on assigning the vehicle control (0.1% DMSO) group a value of 1. Black arrows indicate examples of SR-A or CD36 positivity. *In vivo* foam-cell formation (**B**) and western blot for scavenger receptors in aorta samples (**G**,**H**) are expressed as mean ± SEM. One-way ANOVA was followed by the Holm-Sidak method. Other data were expressed as the median with 25th and 75th percentiles. Kruskal-Wallis test was followed by the Dunn post-hoc test. *P ≤ 0.05; **P ≤ 0.01; ***P ≤ 0.001; C (vehicle control); LanC-LD (low-dose lanatoside C); LanC-HD (high-dose lanatoside C).

Peritoneal macrophages were collected from the different lanatoside C-dose ApoE^–/–^ mice for 12 weeks, and analysed for foam-cell formation with Oil-red-O staining. Our results showed that lanatoside C dose-dependently exacerbated foam-cell formation (Fig. 2A,B). To assess whether lanatoside C promotes atherosclerosis development by inducing SR-A and CD36 in ApoE^–/–^ mice, atherosclerotic lesions were analyzed for SR-A and CD36 expression by immunohistochemistry and immunoblotting. More surprisingly, SR-A and CD36 expression in the cells of the atherosclerotic lesions was markedly enhanced in the lanatoside C-treated ApoE^–/–^ mice (especially in the high-dose group) (Fig. 2C–E). At the same time, the western blotting of the aorta samples also revealed the increased expression of SR-A and CD36 (Fig. 2F–H). In addition, in these progressing atherosclerotic plaques, double-staining for SR-A, CD36 and the macrophage marker CD68 showed that SR-A or CD36 proteins were present in lesional macrophages (Fig. S2, arrows). These data showed that lanatoside C may increase atherosclerosis by upregulation of SR-A and CD36.

### Lanatoside C exacerbates foam-cell formation

**Figure 3:**
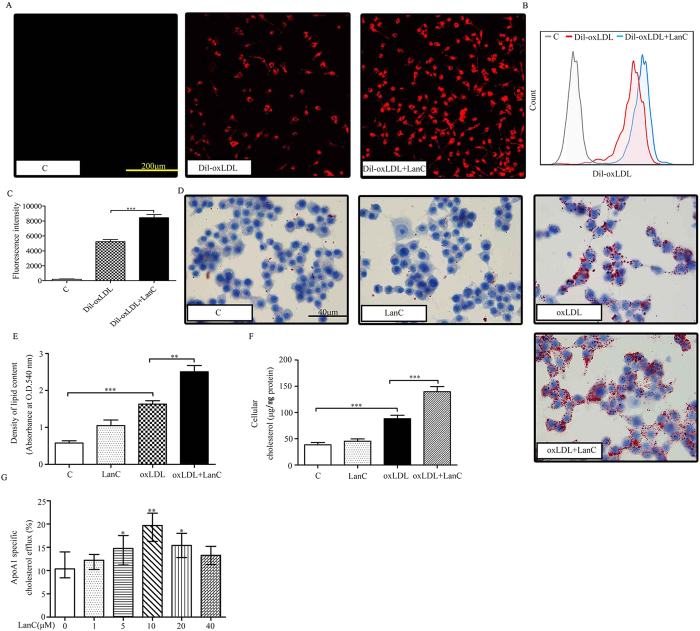
Effects of lanatoside C on macrophage DiI-oxLDL uptake and foam-cell formation *in vitro*. Peritoneal macrophages from untreated mice were incubated with lanatoside C (10 μM) or vehicle control (PBS containing 0.1% DMSO) for 12 h. The cells were then washed twice with PBS and incubated with DiI-oxLDL (10 μg/ml) for 4 h. DiI-oxLDL uptake was assessed by confocal microscopy (**A**) and flow cytometry (**B**,**C**). Treatment of mouse peritoneal macrophages was done with vehicle control (PBS containing 0.1% DMSO), lanatoside C (10 μM) or oxLDL (50 μg/ml) for 24 h. After treatment, the cells were stained with Oil Red O and hematoxylin (**D**). (**E**) The Oil Red O stain was extracted, and the absorbance was measured at 540 nm to measure the amount of lipid contents. (**F**) Cells were prepared and analyzed for cholesterol. (**G**) Macrophages were incubated with the indicated concentrations of lanatoside C (0, 1, 5, 10, 20 and 40 μM) for 12 h, followed by incubation with NBD-cholesterol (1 μg/ml) for an additional 6 h in the presence of lanatoside C. The efflux of cholesterol was initiated by the addition of ApoA-I. Cholesterol efflux was expressed as the percentage of fluorescence in medium relative to the total amount of fluorescence in both cells and medium (*indicates a comparison with the PBS-treated group). ApoA-I-mediated cholesterol efflux (**G**) was expressed as the median with 25th and 75th percentiles. Kruskal-Wallis test was followed by the Dunn post-hoc test. Other data are expressed as mean ± SEM. One-way ANOVA was followed by the Holm-Sidak method in six independent experiments. *P ≤ 0.05; **P ≤ 0.01; ***P ≤ 0.001; C (vehicle control); LanC (lanatoside C).

To further explore the effects of lanatoside C on DiI-oxLDL uptake and foam-cell formation, peritoneal macrophages from 8-week-old male C57BL/6 mice were incubated with lanatoside C (10 μm) for 12 h. The cells were then washed twice with PBS and incubated with DiI-oxLDL (10 μg/ml) for 4 h. We observed that lanatoside C treatment markedly facilitated DiI-oxLDL uptake in macrophages on confocal microscopy (Fig. 3A). Similar results were obtained using flow cytometric analysis of DiI-oxLDL uptake in macrophages (PE-A fluorescence-intensity LanC-added DiI-oxLDL groups, 8153.67 ± 352.8 versus 5245.72 ± 429.84, compared with the DiI-oxLDL-only group) (Fig. 3B,C). Macrophages were loaded with oxLDL (50 μg/ml) in the presence of lanatoside C (10 μM) for 24 h, and Oil Red O staining was performed. We found that lanatoside C significantly accelerated foam-cell formation (Fig. 3D). Similar results were noted when the Oil Red O extracted from the macrophages was measured by spectrophotometry (Fig. 3E). Cellular cholesterol levels were also increased (Fig. 3F). These results indicated that the foam-cell formation was most likely exacerbated atherosclerosis. We subsequently examined the effects of lanatoside C on cholesterol efflux, and observed that lanatoside C significantly promoted ApoA-I-mediated cholesterol efflux (Fig. 3G).

### Lanatoside C regulates the expression of scavenger receptors and cholesterol transporters

**Figure 4:**
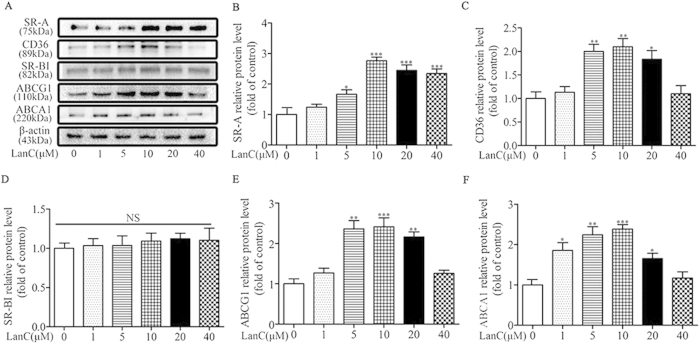
Effects of lanatoside C on the protein-expression levels of the scavenger receptors and the cholesterol transporters in peritoneal macrophages. (**A**) Macrophages from untreated mice were incubated with the indicated concentrations of lanatoside C for 24 h. The cell lysates were subjected to western blot to determine the protein levels of SR-A, CD36, SR-BI, ABCA1, ABCG1 and β-actin. (**B**–**F**) The fold change relative to the control-treated group was used to define the protein levels (after normalization to β-actin) relative to the vehicle-control (PBS containing 0.1% DMSO)-treated group, which was set to 1. All data are expressed as mean ± SEM. One-way ANOVA was followed by the Holm-Sidak method in six independent experiments. *P ≤ 0.05; **P ≤ 0.01; ***P ≤ 0.001 versus vehicle control group; LanC (lanatoside C).

Based on our data showing that lanatoside C promoted oxLDL uptake and the formation of foam cells, we investigated the effects of lanatoside C on the expression of scavenger receptors and cholesterol transporters in the mouse peritoneal macrophages. Treatment with lanatoside C caused a dose-dependent increase in the protein levels of SR-A, CD36, ABCA1 and ABCG1, but not in SR-BI (Fig. 4A–F). Taken together, these data suggest that lanatoside C may promote oxLDL uptake and foam-cell formation by SR-A and CD36.

### Effect of lanatoside C on PPARβ/δ expression in macrophages

**Figure 5:**
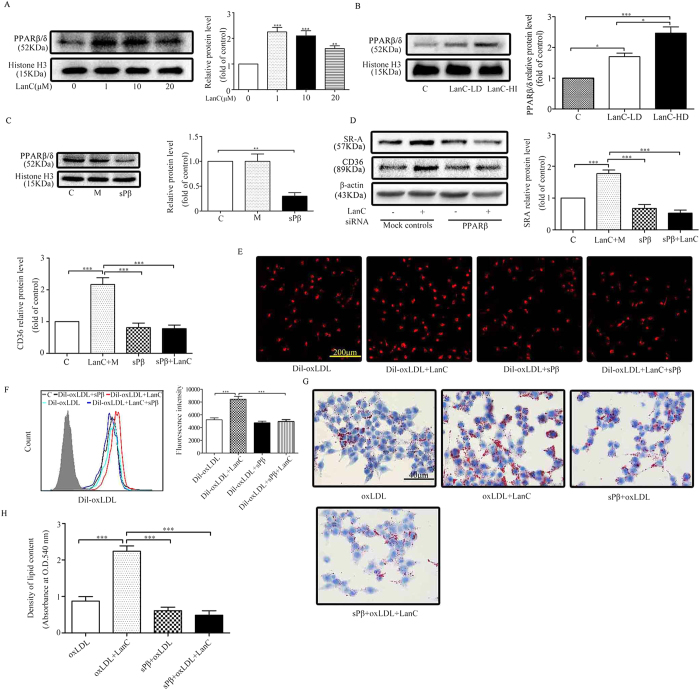
Effects of PPARβ/δ knockdown on oxLDL uptake and foam-cell formation in macrophages. (**A**) Peritoneal macrophages from untreated mice were treated with the indicated concentrations of lanatoside C for 24 h. Then, nuclear protein extracts were isolated and subjected to western blot to determine the nuclear protein levels of PPARβ/δ and histone H3 (compared with the controls). (**B**) Nuclear proteins were isolated and subjected to western blot to determine the nuclear protein levels of PPARβ/δ in the aorta samples (n = 5 per group). (**C**) Macrophages were either transfected with control siRNA as mock controls (M) or with PPARβ/δ siRNA (sPβ) to obtain PPARβ/δ knockdown cells. Western blot was carried out with the specific antibody against PPARβ/δ and histone H3 detected as a loading control. (**D**) Macrophages were transfected with PPARβ/δ siRNA and treated with lanatoside C (10 μM) for an additional 24 h. The cell lysates were subjected to western blot to evaluate the protein levels of SR-A, CD36 and β-actin. The fold change in protein expression in the treated cells relative to the control cells was determined (after normalization to β-actin) based on assigning the vehicle control (PBS containing 0.1% DMSO) group a value of 1. **(E**–**H**) The oxLDL uptake and foam-cell formation were detected in the transfected and control cells. All data are expressed as mean ± SEM of six independent experiments. One-way ANOVA was followed by the Holm-Sidak method. *P ≤ 0.05; **P ≤ 0.01; ***P ≤ 0.001; * indicates versus control. C (vehicle control); LanC-LD (low-dose lanatoside C); LanC-HD (high-dose lanatoside C); LanC (lanatoside C).

To further investigate the mechanism by which lanatoside C regulates scavenger receptor expression, we evaluated the effects of lanatoside C on PPARβ/δ expression in peritoneal macrophages. Lanatoside C increased the expression of nuclear protein PPARβ/δ in a dose-dependent manner (0, 1, 10 and 20 μm) (Fig. 5A). We also obtained the same results from the aorta samples of lanatoside C-treated ApoE^–/–^ mice (Fig. 5B). Furthermore, to explore the transcriptional regulation of PPARβ/δ in lanatoside C-treated peritoneal macrophages, the macrophages were transfected with small interfering RNA (siRNA) (Fig. 5C), and the role of PPARβ/δ as a regulator of cholesterol uptake and foam-cell formation was evaluated. Our results showed that PPARβ/δ siRNA abolished the induction of SR-A and CD36 by lanatoside C (Fig. 5D). The same results were obtained with pharmacological inhibition of PPARβ/δ (Fig. S3). Meanwhile, siRNA further abrogated the increasing effect of lanatoside C on cholesterol uptake (PE-A fluorescence-intensity DiI-oxLDL group, 5038.27 ± 314.02; DiI-oxLDL-added LanC group, 8011.90 ± 542.1; PPARβ/δ siRNA [sPβ]-added DiI-oxLDL group, 4774 ± 293.73; and PPARβ/δ siRNA [sPβ]-added DiI-oxLDL and LanC groups, 4809.29 ± 107.92, respectively) (Fig. 5E,F) and foam-cell formation (Fig. 5G,H). These results imply the essential role of PPARβ/δ in lanatoside C-regulated gene expression of SR-A and CD36, contributing to the upregulation effect of lanatoside C in cholesterol uptake and foam-cell formation.

## Discussion

Atherosclerosis is an inflammatory response of macrophages and lymphocytes to invading pathogenic lipoproteins in the arterial wall, and formation of foam cells by macrophages in the intima is a major hallmark of early-stage atherosclerotic lesions^37^,^38^,^39^. In the present study, we have shown that lanatoside C (especially with the high-dose treatment) significantly increased the development of atherosclerotic lesions in ApoE-knockout mice. The pro-atherogenic effects of lanatoside C appeared to be mediated, at least in part, by markedly aggravated foam-cell formation . These observations served as direct evidence of the pro-atherogenic effects of lanatoside C, which markedly increased SR-A and CD36 expression by upregulation of PPARβ/δ, and promoted lipid uptake and foam-cell formation.

Studies have indicated that foam cells play a major role in plaque formation during atherosclerosis^40^. As a model for a murine atherosclerotic disease model, ApoE^−/−^ mice were used to evaluate the effects of lanatoside C on the development of atherosclerosis. In this mouse model, our results showed that lanatoside C promoted the formation of foam cells in a concentration-dependent manner (Fig. 2A,B). This was also confirmed by *in vitro* studies of mouse peritoneal macrophages (Fig. 3D,E). Lanatoside C caused no changes in plasma total cholesterol, HDL cholesterol, LDL cholesterol or triglyceride levels (Table 1). Based on this data, lanatoside C-promoted atherosclerosis would likely have resulted from foam-cell formation *in vivo*.

Macrophage-derived foam-cell formation is enhanced with relatively increased cholesterol uptake or reduced cholesterol efflux, respectively^40^. Excess uptake of modified forms of LDL by macrophages via scavenger receptors has been well-established as a critical step leading to massive lipid accumulation, foam-cell formation and atherosclerosis. Our *in vitro* foam-cell assay revealed that lanatoside C increased DiI-oxLDL uptake in peritoneal macrophages (Fig. 3A–C). SR-A and CD36 have been centrally implicated in this lipid-uptake process^41^,^42^. To further investigate whether SR-A and CD36 were required for the increased oxLDL uptake and macrophage foam-cell formation, we compared the scavenger receptor expression in macrophages co-cultured with or without lanatoside C. We observed a dose-dependent increase in protein levels of SR-A and CD36 *in vitro* studies (Fig. 4A–C). Surprisingly, our results showed that expression of SR-A and CD36 was also enhanced in atherosclerotic lesions and aorta samples (Fig. 2C–H). These observations suggested that lanatoside C-induced SR-A and CD36 may be a primary cause of increased oxLDL uptake and foam-cell formation. Macrophage lipid homeostasis can be viewed as dynamically regulated by oxidatively modified LDL internalization and cholesterol efflux. Lipid efflux plays a vital role in maintaining homeostasis with the membrane transporters ABCA1, ABCG1 and SR-BI in macrophages^43^,^44^,^45^. Therefore, we examined the regulatory role of lanatoside C and observed augmented protein expression of ABCA1 and ABCG1, but not of SR-BI (Fig. 4A,D–F). This finding indicated that lanatoside C may affect cholesterol efflux. It also confirmed that lanatoside C increased ApoA-I-mediated cholesterol efflux in a dose-dependent manner (Fig. 3G). Lanatoside C treatment exhibited contradictory effects in our study, as it increased foam-cell formation but also promoted the expression of ABCA1, resulting in increased ApoA-I-mediated cholesterol efflux. This finding indicated that the promotion effect of foam-cell formation by lanatoside C may override the effects of ApoA-I-mediated cholesterol efflux.

More importantly, we showed that lanatoside C increased PPARβ/δ expression in peritoneal macrophages (Fig. 5A). Aorta samples also revealed increased nuclear protein PPARβ/δ levels in the ApoE^–/–^ mice treated with lanatoside C (Fig. 5B). Previous studies have demonstrated that PPARβ/δ controls lipid uptake via the class A and class B scavenger receptors (SR-A and CD36), and overexpression of PPARβ/δ promotes lipid accumulation and increases foam-cell formation by SR-A and CD36^30^. The earlier study would fit nicely with the present results regarding the upregulation of SR-A and CD36 by lanatoside C, accompanied by increased nuclear protein PPARβ/δ levels. Moreover, inhibition of upregulation of PPARβ/δ by siRNA abolished the lanatoside C-mediated increased expression of SR-A and CD36 (Fig. 5D). In the functional analysis, inhibition of PPARβ/δ by siRNA resulted in macrophage uptake of Dil-oxLDL that was markedly lower than that of the control group (Fig. 6E,F). In addition, lanatoside C-induced foam-cell formation was significantly reduced by PPARβ/δ siRNA transfection (Fig. 5G,H). Our results suggested that lanatoside C promotes the expression of SR-A and CD36, improves the uptake of oxLDL, and promotes foam-cell formation by upregulation of PPARβ/δ.

In summary, our results provide convincing evidence that lanatoside C aggravates the progression of atherosclerosis in a mouse model of atherosclerosis. These effects appear to be mediated by upregulation of PPARβ/δ, which results in increasing oxLDL uptake and foam-cell formation by SR-A and CD36. The detailed mechanism of lanatoside C-induced PPARβ/δ expression is under investigation. In patients with heart failure or cardiac arrhythmia who are treated with lanatoside C, there may be a risk of the development of atherosclerosis. However, there are significant differences between animals and humans, and further studies are necessary.

## Methods

### Reagents and antibodies

Murine peritoneal macrophages were cultured in DMEM (Gibco, Life Technologies) suppleented with 10% FCS (Gibco, Life Technologies) and 100 U/ml of streptomycin/penicillin. The human copper-oxidized low-density lipoprotein (oxLDL BT910) and DiI-Ox-LDL (BT920) were obtained from Biomedical Technologies. Lanatoside C (L2261), NBD-cholesterol (N2161) and Apolipoprotein A-I (ApoA-I, SRP4693) were obtained from Sigma (St. Louis, Missouri, USA). Anti-ABCA1 (NB400-105), anti-ABCG1 (NB400-132) and anti-SR-BI antibodies (NB400-104) were purchased from Novus Biologicals, Inc. (Littleton, CO). PPARβ/δ siRNA (sc-36306), anti-CD36 (sc-9154), anti-PPARβ/δ (sc-7197), anti-β-actin (sc-130656) and anti-Histone H3 antibodies (sc-10809) were obtained from Santa Cruz Biotechnology Inc (Santa Cruz, California, USA). GSK3787 (188591-46-0) was purchased from Cayman.

### Animals

ApoE^–/–^ mice on a C57BL/6 background were purchased from the Jackson Laboratory and were bred and maintained in the Animal Center of Beijing University. The mice (8 weeks old, 19–22 g) were maintained in a specific pathogen-free facility (Tongji Medical College). The animals were randomly separated into three groups (n = 14 mice/group). The first group, the vehicle control, was injected with the same volume of PBS containing 0.1% DMSO, while the second and third groups received intraperitoneal injections of approximately 20 μg of lanatoside C (low-dose, 1 mg/kg per day) or 40 μg lanatoside C (high-dose, 2 mg/kg per day) and were fed an atherogenic Western-type diet, containing 0.15% cholesterol and 21% fat, for 12 weeks. Blood samples for lipids were collected retro-orbitally on days 0, 30, 60 and 90 of the study. At the end of the 12-week treatment period, on the day of sacrifice, all animals were anaesthetised intraperitoneally with sodium pentobarbital (50 mg/kg), then exsanguinated via a retro-orbital venous puncture under general anaesthesia. The mice were subsequently euthanised by cervical dislocation. Blood samples were obtained from the animals and stored at −80 °C until further analysis. After the animals were sacrificed, their aortic arches were isolated. All experimental procedures and animal care were performed in accordance with the NIH guidelines and approved by the ethics committee on the Care and Use of Laboratory Animals (Science and Technology Department of Hubei Province, China).

### The isolation of peritoneal macrophages

Eight-week-old male C57BL/6 untreated mice, or mice at the end of the 12-week treatment by lanatoside C, were used for each of the experiments. The mice were euthanised by rapid cervical dislocation, and 5 ml of PBS was injected and withdrawn intraperitoneally, after which the samples were centrifuged at 1000 g for 5 min. The cells were resuspended in DMEM containing 10% FBS and 100 U/ml of penicillin/streptomycin for use in subsequent experiments (at a concentration of 1 × 10^6^/ml).

### Transfection of siRNA

PPARβ/δ siRNA were purchased from Santa Cruz Biotechnology. Peritoneal macrophages are 60–80% confluent and wash the cells once with 2 ml transfection medium. The different dose of PPARβ/δ siRNA were used for measuring efficiency of siRNA transfection in our preliminary experiment, our results showed that PPARβ/δ siRNA (100 nM) can obviously reduced PPARβ/δ protein level. Therefore, the final concentration of PPARβ/δ siRNA using 100 nM was transfected by the Lipofectamine 2000 according to the manufacturer’s instructions. Transfected cells were washed in new culture media for use in subsequent experiments.

### Atherosclerosis quantitation and assessment

Mice placed on a western diet for 12 weeks were anaesthetized intraperitoneally with sodium pentobarbital (50 mg/kg), and exsanguinated via a retroorbital venous puncture under general anesthesia, subsequently euthanized by fast cervical dislocation. Aortas were collected from the base of ascending aorta and to the iliac bifurcation, whereas aortic roots with heart were harvested. Aortas for en face were stained with Oil Red O. En face preparations of the entire aortas were dissected and fixed, opened longitudinally and pinned onto black wax plates, after which they were visualized by staining with Oil Red O (Sigma, St. Louis, Mo). The aortic roots were fixed in 4% formaldehyde, processed and embedded in optimum cutting temperature (OCT) compound. The resultant aortic sinus cryosections (7 μm) were stained with Oil Red O and hematoxylin. The mean atherosclerotic areas were calculated from 8 different mice, and 10 serial cryosections/tissue sections for each mouse were evaluated. The total atherosclerotic area for each plaque area measurement from each mouse was used for the calculation. Image-Pro Plus6.703 software (Media Cybernetics) was used for the statistical analysis.

### Immunostaining and immunofluorescence

For histological analysis, the samples were rinsed with PBS and the aortic roots were routinely embedded in OTC compound. The aortic roots were cut into 5 μm serial cryostat sections on the aortic valve plane. Cryosections were fixed in 4% paraformaldehyde for 30 min and rinsed in TBS. Nonspecific sites were blocked using the avidin/biotin blocking kit, followed by 1% BSA (Sigma) and 5% normal goat serum in PBS. Slides were incubated overnight at 4°C with anti-SR-A antibody (1:200, AbD Serotec), anti-CD36 antibody (1:50, Santa Cruz) and anti-CD68 antibody (1:100, Abcam) for macrophages. The slides were then rinsed and incubated with secondary antibody. The processed sections were visualised using an Olympus microscope (IX71; Olympus Corporation, Tokyo, Japan) and a fluorescent microscope (Olympus Microscope BX-51, Japan) or a confocal microscope (Alsi, Nikon). Means were taken from seven different mice, evaluating ten serial cryosections/tissue from each mouse. SR-A and CD36 were quantified by assessing the percent positive area of total plaque for each marker. Image-Pro Plus 6.703 software (Media Cybernetics) was used for the statistical analysis.

### Studies of macrophage foam cell formation

Peritoneal macrophages from untreated mice were cultured overnight on chamber slides (Nalge Nunc International) in DMEM containing 10% FBS and 100 U/ml penicillin/streptomycin. The cells were serum-starved for 12 hours and subsequently treated with vehicle control (PBS contained 0.1% DMSO), lanatoside C (10 μM) or oxLDL (50 μg/ml) for an additional 24 hours. The non-adherent cells were washed away and the adherent cells were collected for Oil-red-O staining or cholesterol measurement. Upon fixation with paraformaldehyde (4%), the cells were stained with 0.3% oil red O for 15 min, Hematoxylin was used as a counterstain, and evaluated via microscopy. The stained cells were eluted with isopropanol, and the supernatant was collected; the OD of the extracts was measured at 540 nm.

### Protein extraction and immunoblotting

For western blot of SR-A, CD36 and PPARβ/δ in the aorta samples, the ApoE^−/−^ mice were sacrified after being fed a Western diet and treated with vehicle control (PBS containing 0.1% DMSO) or lanatoside C (low-dose, 1 mg/kg per day versus high-dose, 2 mg/kg per day) for 12 weeks. The aortas were immediately excised and frozen in liquid nitrogen. After tissue homogenization, cytoplasmic and nuclear protein extraction was subjected to western blot for CD36 and PPARβ/δ. Following treatment, the cells were washed twice with cold PBS. The nuclear and cytoplasmic proteins were extracted using NE-PER Nuclear and Cytoplasmic Extraction Reagents (Thermo Scientific) according to the protocol recommended by the manufacturer. The protein concentration was determined using the Lowry assay. Equal amounts of protein were separated by either 10% or 12% SDS-polyacrylamide (SDS-PAGE) gels and then electrophoretically transferred onto a nylon-enhanced nitrocellulose membrane. The membranes were blocked for 2 h in 5% non-fat powdered milk dissolved in Tween/PBS buffer, then incubated with different dilutions of the indicated primary antibodies at 4 °C overnight. They were then incubated with either anti-mouse or anti-rabbit HRP-conjugated IgG at room temperature for 2 h prior to detection using ECL substrate (Thermo Scientific). Protein expression levels were semiquantitatively analysed using densitometry analysis.

### RNA extraction and real-time RT-PCR

Peritoneal macrophages from untreated mice were treated with PPARβ/δ antagonist (GSK3787 10 μM) 4 h prior to lanatoside C treatment for 24 h. Total RNA was prepared using TRIzol Plus (Takara Biotechnology, Japan), according to the manufacturer’s instructions. RNA purity and concentrations were measured using a spectrophotometer. One microgram of total RNA was reverse-transcribed into cDNA using the RNA PCR Kit (Takara), and the cDNA was used as a template. The sequences of the primers are shown in Table S2. The PCR reaction mixture was prepared using SYBR Green (Takara). The real-time PCR was performed using the Applied Biosystems Step One, and the housekeeping gene GAPDH was used as an internal control for the PCRs. Fold changes in the target gene mRNA were determined using the formula 2^−△△Ct^.

### Cholesterol efflux

Peritoneal macrophages from untreated mice were treated with various concentrations of lanatoside C (0, 1, 5, 10, 20 and 40 μM) or with 0.1% DMSO for 12 h before being equilibrated with NBD-cholesterol (1 μg/ml) for an additional 6 h, in the presence of either or lanatoside C. The NBD-cholesterol–labelled macrophages were washed three times with PBS, then the cells were incubated in DMEM medium containing 0.2% (w/v) fatty acid-free BSA or ApoA-I (10 μg/ml) for 6 h, and finally rinsed twice with PBS. The number of cells and the levels of medium fluorescence-labelled cholesterol were measured using a multi-label counter (PerkinElmer, Waltham, MA, USA). Cholesterol efflux was expressed as a percentage of fluorescence in the medium relative to the total amount of fluorescence.

### Lipid measurement

The plasma levels of total cholesterol, triglycerides, low-density lipoprotein (LDL) and high-density lipoprotein (HDL) cholesterol were measured using chemically modified enzyme-based assay kits (KYOWA MEDEX). The cellular lipids were harvested by sonicating the cells in hexane/isopropanol (3/2, v/v). After removal of the cellular debris via centrifugation at 12,000 g, the supernatant was dried under nitrogen flush. Total cholesterol was determined using an Amplex® Red Cholesterol Assay kit (Life Technologies). Protein levels were determined using the Lowry assay.

### DiI-oxLDL uptake

Peritoneal macrophages were seeded on confocal dishes (Corning, NY) at a density of 5 × 10^5^ cells/ml before being serum starved and pre-incubated with vehicle control (PBS contain 0.1% DMSO), or lanatoside C (10 μM) for 12 h. Then, the cells were incubated with DiI–labeled oxidized LDL for 4 hours. The cells were fixed in phosphate-buffered 4% formalin for 10 min. The cells were analyzed by confocal microscopy. Then, the cells were quantitatively analyzed via flow cytometry.

### Flow cytometry

Macrophages pre-incubated with vehicle control (PBS contain 0.1% DMSO), or lanatoside C (10 μM) for 12 h. Then, the cells were incubated with DiI–labeled oxidized LDL for 4 hours, and washed 3 times with PBS and twice with PBS-2 mg/ml BSA. Thereafter, the cells were detached with trypsin (0.25% trypsin, 0.02% EDTA). The cells were harvested with RPMI 1640/10% FBS, centrifuged at 1,000 rpm for 5 min, washed twice with PBS. Flow cytometry (a total of 10,000 events; Excitation, 514 nm; Emission; 550 nm) was performed on a FACSCalibur (BD Immunocytometry Systems). Data were calculated by subtracting the cell autofluorescence from the fluorescence of the treated samples and expressed as fluorescence intensity. The data were analyzed by FlowJo software (Treestar Inc).

### Lanatoside C plasma measurement

Twenty-four hours after the final injection, blood was collected from the retroorbital vein, mixed and drawn into a capillary tube. The plasma was separated via low-speed centrifugation, collected. Lanatoside C levels were measured using a commercially available Test system kit (Monobind).

### Statistical analysis

Results are expressed as the means ± SEMs when normally distributed. When data did not pass the test for normality, they are presented as median with 25th and 75th percentiles. All data was first evaluated for normal distribution using the Kolmogorov-Smirnov test. When data were normally distributed and group variances were equal, comparisons between 2 groups were performed by the Student t test. The Mann–Whitney rank sum test was used when data were not normally distributed or if group variances were unequal. One-way ANOVA was used for multiple comparisons between ≥3 groups followed by the Holm–Sidak test when data were normally distributed and group variances were equal. When group data were not normally distributed or if group variances were unequal, the Kruskal–Wallis test followed by the Dunn posthoc test was used. P-values < 0.05 were considered statistically significant. All statistical analyses were performed using SPSS software (version 17.0, SPSS Inc, Chicago, IL).

## Additional Information

**How to cite this article**: Shi, H. *et al.* Lanatoside C Promotes Foam Cell Formation and Atherosclerosis. *Sci. Rep.* **6**, 20154; doi: 10.1038/srep20154 (2016).

## Supplementary Material

Supplementary Information

## Figures and Tables

**Table 1:** The effects of lanatoside C treatment on plasma cholesterol levels.

	TC, mg/dL	TG, mg/dL	LDL-C, mg/dL	HDL-C, mg/dL
C				
0 days	310 ± 152.19	155 ± 39.21	192 ± 83.79	114 ± 63.04
30 days	1092 ± 217.31	387 ± 67.91	859 ± 139.45	192 ± 73.41
60 days	1194 ± 417.59	413 ± 93.10	864 ± 104.32	214 ± 83.40
90 days	1205 ± 461.13	420 ± 109.53	870 ± 153.73	221 ± 99.74
LanC-LD				
0 days	319 ± 97.35	160 ± 54.91	199 ± 63.30	124 ± 64.94
30 days	1104 ± 393.73	397 ± 83.20	855 ± 103.20	204 ± 104.63
60 days	1091 ± 421.32	419 ± 129.0	862 ± 130.03	217 ± 94.41
90 days	1291 ± 379.92	427 ± 129.2	865 ± 129.43	230 ± 103.90
LanC-HD				
0 days	303 ± 115.73	166 ± 52.31	208 ± 49.02	119 ± 89.23
30 days	1102 ± 321.95	391 ± 109.73	860 ± 136.32	210 ± 59.30
60 days	1121 ± 532.84	412 ± 94.24	867 ± 149.30	213 ± 105.32
90 days	1210 ± 510.62	428 ± 102.34	872 ± 152.42	224 ± 94.82

The data are expressed as mean ± SEM in four independent experiments. Repeated-measures ANOVA was followed by the post-hoc test. *P ≤ 0.05; **P ≤ 0.01; ***P ≤ 0.001; C, n = 8; LanC-LD, n = 8; LanC-HD, n = 8; TC (total cholesterol); TG (triglycerides); LDL-C (LDL cholesterol); HDL-C (HDL cholesterol); C (vehicle control); LanC-LD (low-dose lanatoside C); LanC-HD (high-dose lanatoside C).
