# Supplementary material for: Lanatoside C Promotes Foam Cell Formation and Atherosclerosis
Source: Sci Rep. 2016 Jan 29;6:20154. doi: 10.1038/srep20154 (PMC4731744; doi:10.1038/srep20154)
Supplement: Supplementary Information [file srep20154-s1.doc]

**Lanatoside C** [**Promotes Foam Cell Formation and Atherosclerosis**](http://circ.ahajournals.org/content/106/22/2767.short)

Huairui Shi;^1^ Xiaobo Mao;^1^ Yucheng Zhong;^1^ Yuzhou Liu; Xiaoqi Zhao; Kunwu Yu; Ruirui Zhu; Yuzhen Wei; Jianghao Zhu; Haitao Sun; Yi Mao; Qiutang Zeng;^*^


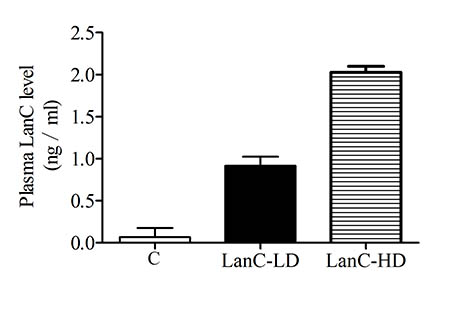


**Fig S1.** Plasma levels of lanatoside C. Plasma levels of lanatoside C in mice treated via i.p. injection of lanatoside C (n=14 each). The data are expressed as the means ± SEMs of three independent experiments. C (vehicle control); LanC-LD (low dose lanatoside C); LanC -HD (High dose lanatoside C); LanC (Lanatoside C).


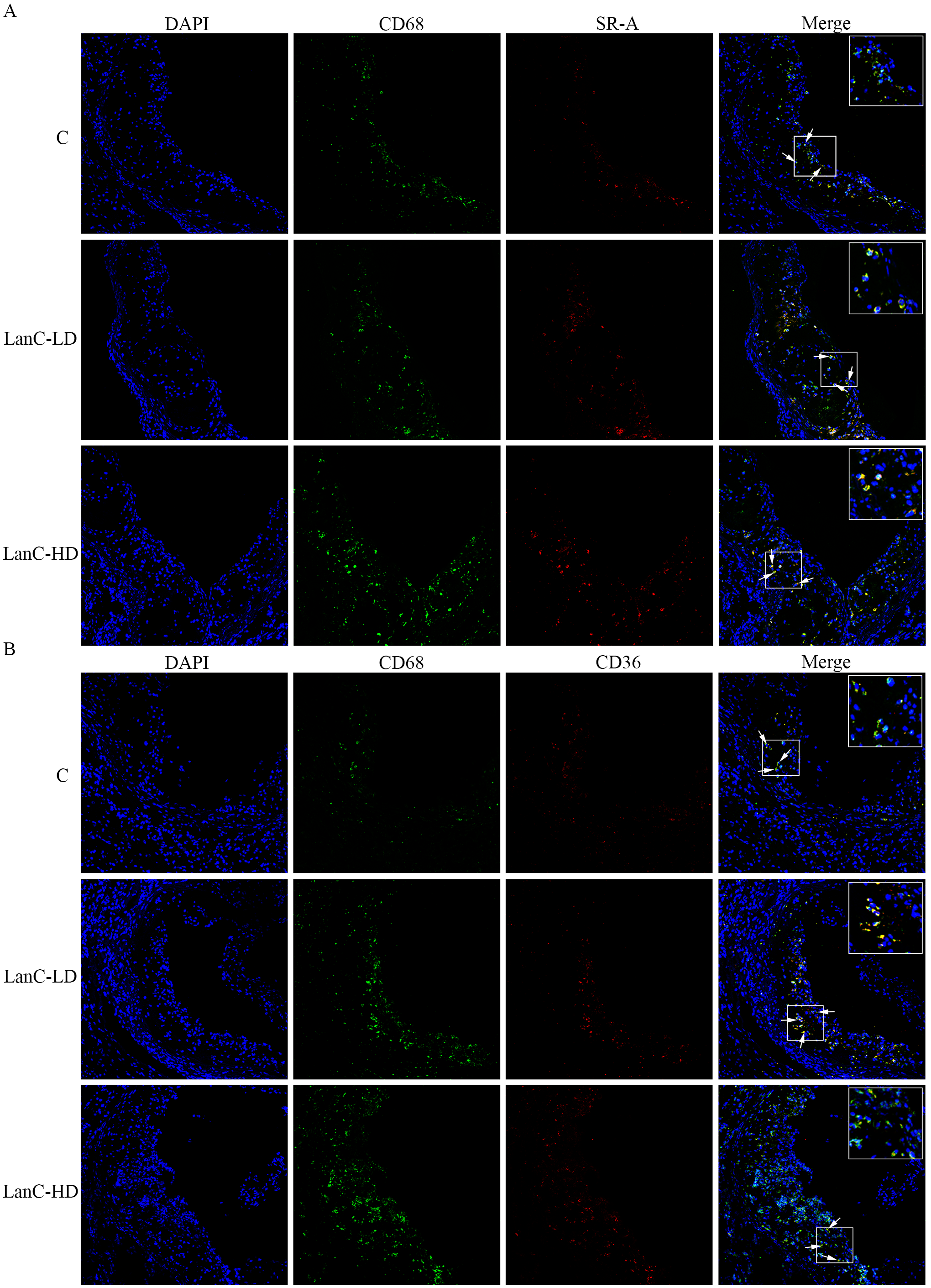
**Fig S2.** SR-A and CD36 are expressed by macrophages in advanced atherosclerotic plaques. Immunofluorescence staining of macrophage CD68 (green), DNA (4',6-diamidino-2-phenylindole [DAPI], blue) and (A) SR-A (red) or (B) CD36 (red) in aortic root atherosclerotic plaques. Areas of colocalization are shown in yellow in the merged image (arrows). C (vehicle control); LanC-LD (low dose lanatoside C); LanC -HD (High dose lanatoside C); LanC (Lanatoside C).

**
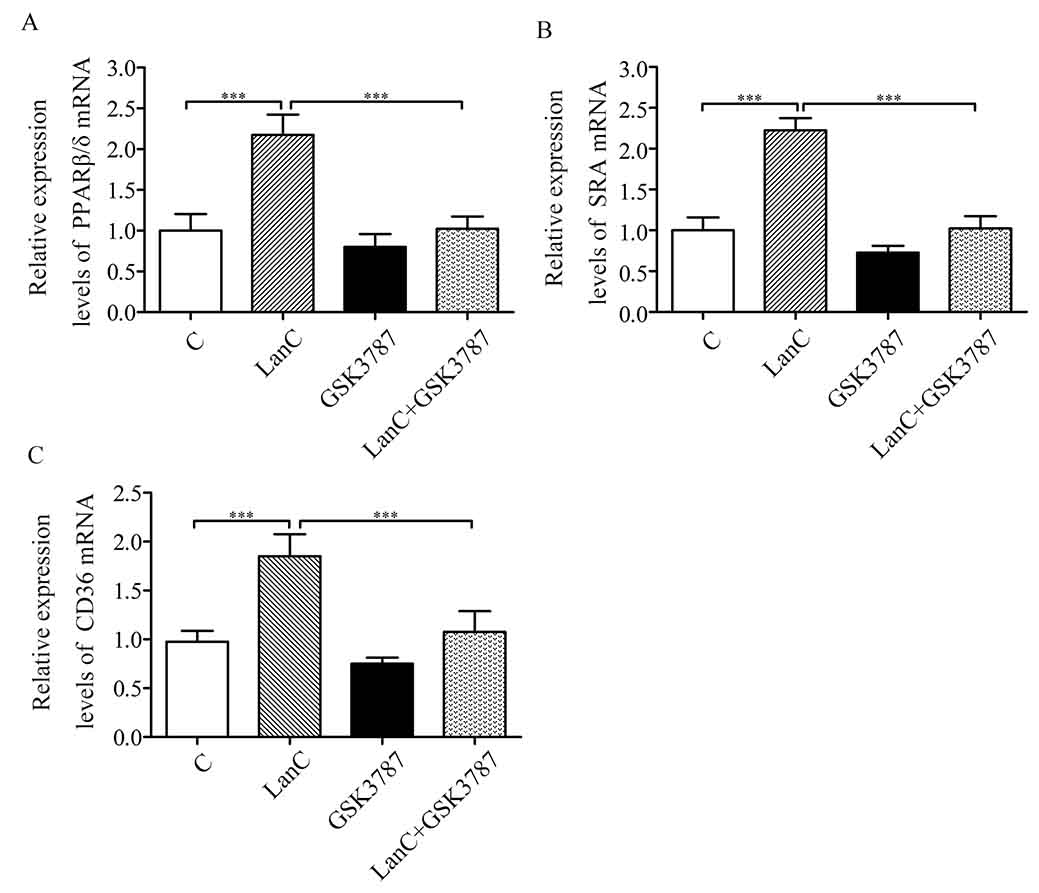
**

**Fig S3.** Effects of lanatoside C on modulation of SR-A and CD36 expression by pharmacological inhibition of PPARβ/δ. Macrophages were pre-incubated with a PPARβ antagonist (GSK3787 10 μM) for 4 hours followed by treatment with PBS, Lanatoside C (10 μM) for an additional 24 hours. (A) PPARβ/δ, (B) SR-A and (C) CD36 expression was determined by RT-PCR analysis. The data are expressed as the means ± SEMs, 1-way ANOVA followed by the Holm-Sidak test method, at five independent experiments, *P≤0.05; **P≤0.01; ***P≤0.001. C (vehicle control); Lanatoside C (LanC).

| 2 mg/kg per day  3 mg/kg per day  4 mg/kg per day | Not obvious abnormalities clinical symptoms  Tachypnea as well as no movement  Convulsions and all mice died within 1h | 0/6  2/6  6/6 |
| --- | --- | --- |

**Table S1.** A higher dose of lanatoside C can increase the risk of a toxic reaction and death.

ApoE-/- mice were separated randomly into 3 groups (n=6 mice/group). The different doses of lanatoside C were given by intraperitoneal injection and observed for 48 h, the obvious abnormalities clinical symptoms and the mortality was noted.

**Table S2** Real time RT-PCR primer sequences.

| PPARβ/δ sense | GCTGCTGCAGAAGATGGCA |
| --- | --- |
| PPARβ/δ antisense | CACTGCATCATCTGGGCATG |
| SR-A sense | TGGTCCACCTGGTGCTCC |
| SR-A antisense | ACCTCCAGGGAAGCCAATTT |
| CD36 sense | CAGTTGGAGACCTGCTTATCC |
| CD36 antisense | GCGTCCTGGGTTACATTTTC |
| GAPDH sense | AGCAATGCCTCCTGCACCACCAAC |
| GAPDH antisense | CCGGAGGGGCCATCCACAGTCT |
